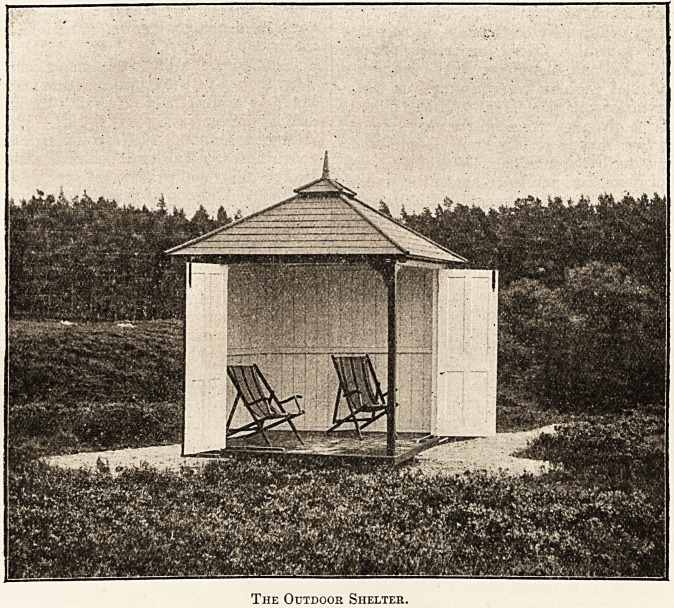# Notes on Sanatorium Management

**Published:** 1910-06-25

**Authors:** 


### SPECIAL ARTICLE.

### NOTES ON SANATORIUM MANAGEMENT.

By A SANATORIUM SUPERINTENDENT.

Existing public sanatoria may be divided into two groups—specially built, and the reverse. The chief examples of the former are the King Edward VII. Sanatorium at Midhurst, the Crossley Sanatorium near Manchester, the Frimley Sanatorium, and the Northwood branch of the Mount Vernon Hospital for Consumption, London, all of them large permanent buildings with about 100 beds each, and all, especially the first, and perhaps excepting the third, most expensively constructed. The newer type, or sub-type, of this class, designed to answer the objections of expense and of length of time in erection, is represented by structures like those at Benenden, in Kent, and Barrasford, in Northumberland. In construction they may be called temporary-permanent, but they have their respective patients all under one roof, as in the institutions above mentioned. Coming to the second group, that of sanatorium buildings provided by adapting a country house or similar existing erection and making small additions thereto, we find a plan which has been adopted, for instance, by the Birmingham Corporation, on an estate near Cheltenham, and by the Durham county branch of the National Association for the Prevention of Tuberculosis. Usually speaking, in this class the administration is located in the house, and a good many of the patients are lodged in out-buildings.

The Outdoor Shelter.

### The Staff.

It is important to premise so much at least in treating of the subject under notice, because both the size of the institution and the design of its accommodation condition the management to a considerable extent. The larger the institution, the more economical, of course, will be its ratio of staff to patients. With a temporary building more provision for upkeep is necessary than with a permanent one. And if the same standard of nursing and supervision—things which vary much in quality in different sanatoriums—is to be maintained, then more nurses will be required to look after patients dotted about in huts and pavilions than after those in one main building. But, taking a 50-bed sanatorium of special construction as being a fairly common example, one may say that the staff required will be a resident medical officer, a matron, and four nurses, one of which last should be fairly senior and fully trained. It must be remembered that in a sanatorium where phthisical pyrexia

(almost universal in incoming patients, for instance) is properly treated, about a third of the whole number are bed-patients. This necessitates a full nursing staff, if not a very highly trained one. As stated, it will be sufficient if the charge nurse is assisted by three probationers, since, in public sanatoriums at any rate, bad cases are usually sent away before they become very ill, and, moreover, very advanced ones are not admitted. The nursing work is thus mainly concerned with bed-making and taking meals to and performing other requisite offices for bed-patients. Nowadays patients nearly always chart their temperatures themselves. No night nurse is required, but the day nurses should sleep at different parts of the building, and the patients' bells must ring in their rooms. If a run of bad cases is encountered temporary extra help can be summoned from the nearest large town. There should be eight or nine maids and a cook. If a private steam laundry is provided for the use of the institution, as will usually be the case, since sanatoriums are generally placed in rather remote country districts, and, moreover, public laundries are often shy of washing for consumptives, then two laundrymaids are called for. The engineer, who should be given a cottage on the premises, so that his services may be readily available in case of mishap, must have thorough knowledge of electrical work and of plumbing, and be competent to undertake all repairs in these departments. £100 a year is not too much for a good engineer. A stoker can be picked up locally and trained by him.

### The Question of Graduated Labour.

The dimensions of the rest of the male subordinate staff depend upon the amount of work which can be expected from the convalescent patients. It must be remembered that the Frimley Sanatorium, whence emanated the now well-known system of "graduated labour," draws picked patients from the extensive wards of the Brompton Hospital for Consumption. Nothing like the same percentage of those who, to use the concise German expression, are "arbeitsfähig," can be expected from a provincial city, even if there exists there a special hospital for the disease. Then many institutions have to depend in part upon paying patients, who may object to a routine of work; and probably the facts yet ascertained are not a warrant for recommending it to them as a therapeutic measure. Though not generally known, it is nevertheless a fact that the occurrence in consumptives of "auto-inoculation" (in Sir Almroth Wright's sense of the word) was first described from examination of patients taking the ordinary Nordrach routine of walking exercise. What probably will constitute the verdict on "graduated labour" of the profession ten years hence is that it forms an indispensable means of preparing manual workers for earning a living after discharge from the sanatorium, and also a help to economical management of these institutions and the maintenance of order in them; but that, therapeutically, it is inferior to simple walking. There must be a porter to take charge of the sputum disposal and disinfection of rooms, etc., with a boy to clean boots. If the male patients work six hours a day they can manage a vegetable garden, which will keep the institution in greenstuff; but the porter should have some knowledge of gardening and be able to direct them. In the winter other work can be found. The better plan in the interests of the patients is, however, to give them work according to their different occupations, and for the furtherance of this a joiner's shop is useful. In a building of more or less temporary construction a joiner with some knowledge of house-painting will be found well worth his wages. With the help of patients all the periodical painting and distempering can be undertaken, and shelters, gates, and palings provided for the development of the grounds, which will often have had to be left unfinished at the opening of the institution for lack of funds and from the desire to get the accommodation ready as soon as possible. Repairs also can be done speedily; and open-air treatment generally means a good deal of glaziery work in mending windows. The interior design of the sanatorium, too, is likely to need modifications and additions. A lift to take meals from the ground floor to bed-patients upstairs may not be found, but it is a great help in serving the food hot and saving the nurses heavy work. It is probably cheaper for any but the largest sanatoriums to hire conveyances. The coal contract will include delivery. Pigs and poultry are very useful, not only as food but also in furnishing manure, the want of which may be felt if there is no stable.

### The Superintendent and Matron.

The resident doctor, matron, and possibly also the engineer, will report monthly to a house committee. Preferably the Secretary of this body should be honorary, and a medical man, in which case there is every chance of a good understanding being promoted between the committee and the resident staff. To the resident medical officer is confided the power of dismissing patients for breach of rules, and often, too, any of the male subordinate staff. In all such cases he has to report his action at once to the committee. He undertakes the general administrative work of the place, supervises the male staff, and is in sole control of the patients. The housekeeping arrangements and accounts, direction of the female domestics, and engaging of nurses fall to the matron, who must be well up in housekeeping and equal to maintaining strict discipline among her servants, and also possess a fair knowledge of cookery, general as well as invalid.

### The Commissariat.

That part of sanatorium management connected with the food-supply is, indeed, most important, as will be seen from the following figures, which relate in round numbers to actual expenditure in catering for an average of sixty to seventy persons (patients and staff):—

| | Weekly cost per head. s. | d. |
|---|---|---|
| Butcher's meat | 4 | 4 |
| Groceries (including potatoes) | 3 | 8 |
| Milk, eggs, and cream | 2 | 11 |
| Fish and poultry | 0 | 8 |
| Bread | 0 | 5½ |

In this case some few of the patients were private ones. A practice which has come into vogue at some purely working-class sanatoriums is to replace butcher's meat two or three times a week by cheese or leguminous foods, not only for the sake of economy but in order to get the patient used to a diet the cost of which will not be beyond his means when he leaves the institution. Clearly local custom must be considered before doing this, and the plan, though tempting from the way in which it enables the bills to be cut down, is much more suitable for an indigent rural population than in the case of industrial districts, where wages and the standard of living rule higher. Consumptives, be it remembered, too, are more likely than ordinary hospital patients to complain of their food, although their *menu* is considerably the more attractive one.

A local butcher will nearly always have the contract for the meat, and as losing it would obviously mean to him a very serious loss in income, he can easily be kept up to the mark; the same holds good of the milk-supply, and, of course, a dairy which serves a large institution has to fulfil certain preliminary legal requirements. A necessary kitchen-fitting is a metal "hot plate," to carry a couple of joints, for carving on, and fitted to hold the requisite number of plates and dishes. "Steamers" and a steam-fitted sink for washing-up in are almost essential, whilst such labour-saving devices as a bread-cutting and potato-peeling machine repay their cost. The medical officer should preside at meals, not only for medical reasons but also in order to secure that perfect order prevails.

### Discipline.

Mention of this introduces another point almost as important as the food-supply, and that is the tactful but strict supervision necessary to preclude the chance of any familiarity occurring between male and female patients, or between male patients and the nurses or domestics.

Consumption is a disease of young people, and sanatorium treatment, with the high feeding and abundant rest and leisure which it involves, is by no means an anaphrodisiac. Consequently any lack of discipline will encourage the beginnings of what may end in serious trouble and a scandal. Constant quiet vigilance on the part of the resident medical officer, the matron, and the charge nurse is called for, and cannot be replaced by even the showiest and most elaborate of routines. It is one of the many advantages of a specially built sanatorium that the construction of the building provides for sharp separation of the sexes. Their respective exercise grounds must be as clearly marked off as their quarters, either by means of a dividing fence or else by observing certain times for men and women in certain areas. A little consultation between doctor and matron will readily suggest measures by which opportunities for conversation between male patients and the maids are reduced to a harmless minimum. Anyone with much resident experience of these institutions will vouch for the necessity for care in this matter.

Private patients, too, are best kept separate from municipal ones. The theory may be to treat a married man with an income of three to four hundred pounds a year just like a young labourer sent in from the workhouse infirmary, but it is hardly possible to put this theory rigidly into practice. No doubt in every institution for the sick there are some surprising social juxtapositions, but if private patients in a sanatorium are lodged on a separate floor and have their meals separately, there will be a greater flow of them seeking for admission, and the medical officer's work will be simplified. The point is worth attention because nowadays, when sanatoriums have multiplied so fast, the supply of paying patients is none too abundant, and, as previously mentioned, the allotting of a few beds to paying patients may enable a public sanatorium to make ends meet which otherwise could not do so.

### Some Conclusions.

The above are some of the material details of sanatorium management which most need mention. But a thoroughly well working institution, whatever its conditions and dimensions and finances, cannot be got without a certain spirit pervading the place, a spirit to be inspired by the medical officer at its head, and by him only.

A sanatorium is not a hospital, where very many, in their varying degree, participate in the medical work and in the everyday routine of management. Sanatorium patients are much less acutely ill and much more able-bodied than hospital patients, and their whole manner of life has to be controlled absolutely. For some such reasons the methods of the British nursing system, praiseworthy in many respects as they are, are out of place in the direction of a sanatorium. Meticulous spick-and-span-ness cannot be maintained there at a reasonable cost, any more than it can in a convalescent home; neither does it need to be, or the consumptive's day to be begun, divided, and rounded off with hymn-singing. But there must be a substitute for these simple means towards order and decorum. Long ago now Dettweiler claimed that the medical director of a sanatorium must be an exceptional man. Whether he often is, at any rate in this country, is another matter; but there can be no doubt far the best and most cheerful and hopeful adherence to routine is obtained where patients are strongly impressed by some exceptional good trait—by the uncommon strength of character of Walther of Nordrach, by a born organiser like Dr. Paterson, by marked intellectual and artistic tastes as in the case of the head of a private institution it is naturally not fair to mention. This influence, aided by that of the *genius loci*, to which Professor Osler has drawn attention, cannot at best, one thinks, affect very greatly the predestination of the grim issue—recovery or death; but it may hasten the one and retard the other, and in any case is stronger than aught else in bringing the patient to observe that way of life his particular physician chooses for him—which, surely, is the whole purpose of each sanatorium.

## Figures and Tables

**Figure f1:**